# Evaluation of interleukin‐1 and interleukin‐6 receptor antagonists in a murine model of acute lung injury

**DOI:** 10.1113/EP091682

**Published:** 2024-04-09

**Authors:** Émilie Meunier, Mélissa Aubin vega, Damien Adam, Anik Privé, Mohammad Ali Mohammad Nezhady, Isabelle Lahaie, Christiane Quiniou, Sylvain Chemtob, Emmanuelle Brochiero

**Affiliations:** ^1^ Centre de Recherche du Centre hospitalier de l'Université de Montréal (CRCHUM) Montréal Québec Canada; ^2^ Département de Médecine Université de Montréal Montréal Québec Canada; ^3^ Centre de recherche du Centre hospitalier Universitaire Sainte‐Justine Montréal Québec Canada; ^4^ Département de pédiatrie Université de Montréal Montréal Québec Canada

**Keywords:** acute respiratory distress syndrome (ARDS), bleomycin, IL‐1, IL‐6, inflammatory response, Kineret, receptor antagonists, tocilizumab

## Abstract

The acute exudative phase of acute respiratory distress syndrome (ARDS), a severe form of respiratory failure, is characterized by alveolar damage, pulmonary oedema, and an exacerbated inflammatory response. There is no effective treatment for this condition, but based on the major contribution of inflammation, anti‐inflammatory strategies have been evaluated in animal models and clinical trials, with conflicting results. In COVID‐19 ARDS patients, interleukin (IL)‐1 and IL‐6 receptor antagonists (IL‐1Ra and IL‐6Ra, kineret and tocilizumab, respectively) have shown some efficacy. Moreover, we have previously developed novel peptides modulating IL‐1R and IL‐6R activity (rytvela and HSJ633, respectively) while preserving immune vigilance and cytoprotective pathways. We aimed to assess the efficacy of these novel IL‐1Ra and IL‐6Ra, compared to commercially available drugs (kineret, tocilizumab) during the exudative phase (day 7) of bleomycin‐induced acute lung injury (ALI) in mice. Our results first showed that none of the IL‐1Ra and IL‐6Ra compounds attenuated bleomycin‐induced weight loss and venous $P_{CO_{2}}$ increase. Histological analyses and lung water content measurements also showed that these drugs did not improve lung injury scores or pulmonary oedema, after the bleomycin challenge. Finally, IL‐1Ra and IL‐6Ra failed to alleviate the inflammatory status of the mice, as indicated by cytokine levels and alveolar neutrophil infiltration. Altogether, these results indicate a lack of beneficial effects of IL‐1R and IL‐6R antagonists on key parameters of ALI in the bleomycin mouse model.

## Abstract (highlights)

- **What is the central question of this study?**
  There is no effective pharmacological treatment for the acute respiratory distress syndrome (ARDS), which has an important inflammatory component: what is the efficacy of novel interleukin‐1/6 receptor antagonists, compared to commercially available antagonists, for resolution of ARDS parameters in vivo?
- **What is the main finding and its importance?**
  The novel and commercial antagonists failed to improve key parameters of the acute phase of ARDS in an animal model of severe acute lung injury. Complementary/alternative strategies favouring epithelial repair or oedema resorption are needed to improve the resolution of ARDS, after severe alveolar damage.

## 1 INTRODUCTION

Acute respiratory distress syndrome (ARDS) (Matthay et al., 2024; Ranieri et al., 2012), a severe form of respiratory failure, is a leading cause of intensive care unit admissions and has a high mortality rate (Bellani et al., 2016). ARDS can be caused by either direct lung insults, for example, following bacterial or viral infections, or indirect injuries, such as sepsis (Bellani et al., 2016; Matthay et al., 2024, 2019). Despite the heterogeneity of ARDS, the acute exudative phase is commonly characterized by extensive alveolar and endothelial damage, leading to pulmonary lung oedema flooding and an exacerbated inflammatory response. The latter is manifested by an increase in pulmonary and systemic levels of pro‐inflammatory cytokines (e.g., interleukin (IL)‐1β, IL‐6, IL‐8, tumour necrosis factor α (TNF‐α)) and neutrophil infiltration into the lungs, both of which amplify alveolar damage and pulmonary oedema (Butt et al., 2016; Matthay et al., 2019; Monahan, 2013). Rapid resolution of this reversible acute phase is critical to the resolution of ARDS before the development and establishment of pulmonary fibrosis, ultimately leading to respiratory or multi‐organ failure (Bosch et al., 2022; Chesnutt et al., 1997; Ketcham et al., 2020; Ware & Matthay, 2000).

Although the management of ARDS has improved over the years (with interventions such as protective mechanical ventilation, fluid management and/or prone positioning) (Griffiths et al., 2019; Papazian et al., 2019), there is still no effective pharmacological treatment for this condition and its mortality rate (30–45%) remains unacceptably high (Azoulay et al., 2018; Bellani et al., 2016; Fan et al., 2018; Fitzgerald et al., 2014; Matthay et al., 2019; Pais et al., 2018). Therefore, the development of new therapeutic strategies that could improve the resolution of ARDS is of critical importance (Fitzgerald et al., 2014). Due to the major contribution of inflammation in the pathophysiology of ARDS (Butt et al., 2016; Matthay et al., 2019; Monahan, 2013), several clinical trials (Fadanni & Calixto, 2023; Fan et al., 2018; Foster, 2010; Kimura et al., 2016; Meduri et al., 2016; Standiford & Ward, 2016; Steinberg et al., 2006; Tongyoo et al., 2016; Yang et al., 2017; Yehya et al., 2015) and experimental studies in animal models of acute lung injury (ALI) (Aubin Vega et al., 2019; Chen et al., 2006; Engel et al., 2015; Hegeman et al., 2013; Leite‐Junior et al., 2008; X.‐Q. Wang et al., 2008; T. Xu et al., 2009; Yubero et al., 2012) have investigated the efficacy of anti‐inflammatory therapies, particularly with systemic corticosteroids. However, the available evidence on the benefit of glucocorticoids is conflicting, and their use in ARDS remains controversial (Bein et al., 2016; Bihari et al., 2016; Bos et al., 2018; Meduri & Siemieniuk, 2017; Seam & Suffredini, 2016; Sweeney & McAuley, 2017; Thompson & Ranieri, 2016).

Treatments targeting specific signalling pathways of the inflammatory response, including antagonists of the IL‐6 receptor (IL‐6Ra, e.g., tocilizumab (Actemra)) and the IL‐1 receptor (IL‐1Ra, e.g., kineret (anakinra)), have also been developed. Tocilizumab was found to be moderately effective in COVID‐19‐related ARDS (Elahi et al., 2022; Felsenstein et al., 2020; Peng et al., 2022; Recovery Collaborative Group, 2021) and this drug was recently approved by the FDA and Health Canada in a subset of hospitalized, ventilated COVID‐19 patients (Food & Drug Administration HHS, 2021; Health Canada, 2022). Although the outcomes of Kineret in COVID‐19 ARDS are more mitigated (Felsenstein et al., 2020; Iglesias‐Julián et al., 2020; Kyriazopoulou et al., 2021; Shang et al., 2023), an emergency use authorization was granted by the FDA for hospitalized COVID‐19 patients at high risk of severe respiratory failure (Food & Drug Administration HHS, 2023). However, the use of Kineret and tocilizumab in non‐COVID‐19 ARDS is still limited due to both their potential immunosuppressive properties and the lack of consensus on the benefit of these antagonists in experimental studies (Engeroff et al., 2022; Gu et al., 2021; Sarıoğlu et al., 2021; Terzi et al., 2022; X. Wang et al., 2021; Zhu et al., 2023).

We have previously designed a small stable (D‐) peptide antagonist (rytvela) that binds to the IL‐1R and biases signalling by blocking both the mitogen‐activated protein kinase (MAPK) and RhoK pathways, while preserving nuclear factor κB (NF‐κB), a key factor in immune vigilance (Quiniou et al., 2008). This peptide was also found to be more effective than its commercial counterpart (Kineret) in various murine models (lipopolysaccharide (LPS), lipoteichoic acid (LTA), IL‐1β‐induced preterm birth, and oxygen‐induced retinopathy of prematurity) (Habelrih et al., 2023; Nadeau‐Vallée et al., 2017, 2015; Quiniou et al., 2008; Sayah et al., 2020). We have also designed an IL‐6R antagonist (HSJ633, with the amino acid sequence VRKFQNSPA) that inhibits signal transducer and activator of transcription 3 (STAT3) while preserving the cytoprotective Akt pathway and shows promising preliminary results in a murine LPS‐induced preterm birth model (unpublished data). However, none of these molecules have been tested in an adult model of induced acute lung injury.

The aim of this study was to evaluate the efficacy of the designed peptide antagonists rytvela and HSJ633, compared to the commercially available antagonists Kineret and tocilizumab, in a mouse model of ALI. We opted for the well‐established model of bleomycin‐induced lung injury and focused our study on the acute phase at day 7 (D7). Although no single animal model perfectly recapitulates all the characteristics of ARDS in humans, the bleomycin‐induced model in its acute phase (at D7) exhibits key pathological features to qualify as an experimental ALI model (as defined by two official American Thoracic Society reports) (Kulkarni et al., 2022; Matute‐Bello et al., 2011), with histological evidence of tissue injury (evolving over time), alteration of the alveolar–capillary barrier (leading to pulmonary oedema flooding), the presence of an inflammatory response (neutrophil infiltration and cytokine production), and physiological dysfunction (with decline in lung function), as shown in our previous studies (Aubin Vega et al., 2021, 2019, 2023, 2024). The effects of the IL‐1R and IL‐6R antagonists were evaluated by assessing the general and pulmonary condition of the mice, lung injury scores, pulmonary oedema formation, and inflammatory response on D7 after bleomycin challenge. Our results showed that none of the compounds had a significant beneficial effect on these parameters in this ALI animal model.

## 2 METHODS

### 2.1 Ethical approval

All animal procedures were approved by the Institutional Animal Protection Committee (CIPA, approval reference no. CM20028EBs) of the Centre de recherche du Centre hospitalier de l'Université de Montréal (CRCHUM), in accordance with the guidelines of the Canadian Council on Animal Care (CCAC). The investigators are aware of the ethical principles under which the *Experimental Physiology* operates and confirm that all experiments comply with the animal ethic principles and standards of *Experimental Physiology*.

### 2.2 Animal care

Wild‐type C57BL/6 mice, originally purchased from Charles River Laboratories (Laval, QC, Canada), were maintained by breeding at the CRCHUM animal care facility from our mouse colony, which was backcrossed every 10 generations. Mice were housed under standard humidity and light conditions (12:12 h light–dark cycles) and had free access to a standard mouse chow and water ad libitum.

### 2.3 Bleomycin‐induced lung injury model

**FIGURE 1:**
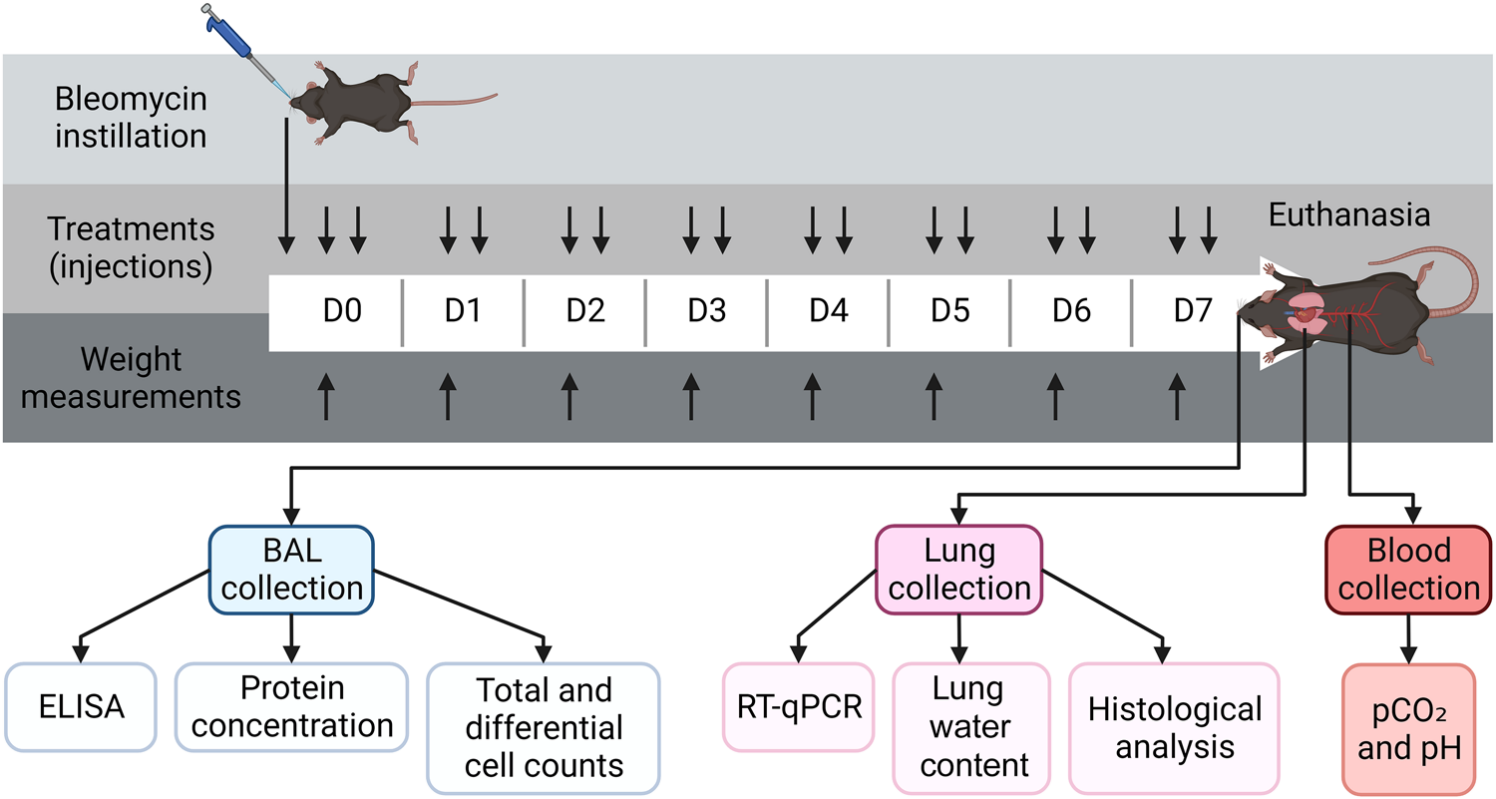
Schematic representation of the experimental protocol, with the time points for the instillation of bleomycin, treatments with receptor antagonists, and subsequent outcome measurements. Created with BioRender.com.

Experiments were conducted on a total of 238 (6‐ to 13‐week‐old male and female) mice, weighing 14.5–33.1 g, randomized into control (phosphate‐buffered saline (PBS)) and bleomycin (Bleo) groups (matched for age, weight and sex). Specifically, mice were anaesthetized with isoflurane (4%) prior to intranasal (i.n.) instillation of 50 μL of PBS or Bleo (3 U/kg; MaynePharma Inc, Raleigh, NC, USA) on day 0 (D0). This route of administration was chosen over the intratracheal technique because it is non‐invasive, rapid (requiring only a few seconds of isoflurane anaesthesia from which the animals regain consciousness very quickly), and spontaneous nasal aspiration allows for a more homogeneous distribution of the administered fluid throughout the lungs. The dose of 3 U/kg of Bleo was chosen to induce severe lung injury without any mortality. Follow‐up of mice for endpoint monitoring included measurement of weight loss, respiratory failure, prostration, uncontrollable pain and dehydration according to the procedure approved by the CRCHUM Institutional Animal Care Committee. There were no animals in the study that required euthanasia due to endpoint attainment. However, some animals were excluded prior to outcome measurements due to technical problems (e.g., inability to retrieve a sufficient volume of bronchoalveolar lavage (BAL)). All animals that underwent outcome measurements were included in the analyses. As detailed in the figure legends, the number of mice varied depending on the parameters that were measured during different sets of experiments. For example, histological analyses, lung water content measurements and BAL had to be performed on different animals. A schematic representation of the experimental protocol, with the time points for bleomycin instillation and treatments with the receptor antagonists, and subsequent outcome measurements described below, is shown in Figure 1.

### 2.4 Treatments with IL‐1β and IL‐6 receptor antagonists

Mice from the control group (i.n. with PBS) were injected subcutaneously (s.c., 200 μL) with saline (0.9%, vehicle, twice daily, from day 0 to day 6; Saline‐PBS), while mice from the bleomycin group (i.n. with Bleo) were randomly divided into five experimental subgroups with the following subcutaneous injections (200 μL, twice daily, from day 0 to day 6): saline (0.9%; Saline‐Bleo), Kineret (Amgen Canada Inc., Mississauga, ON, CA; 12.5 mg/kg/injection; KIN‐Bleo), rytvela (property of the Université de Montréal, 1 mg/kg/injection; Ryt‐Bleo), tocilizumab (Genentech, South San Francisco, CA, USA; 10 mg/kg/injection; TCZ‐Bleo), HSJ633 (property of the Université de Montréal; 1 mg/kg/injection; 633‐Bleo) or a combination of rytvela and HSJ633 (1 mg/kg/injection of each compound; Ryt+633‐Bleo). The doses used were determined based on previous in vivo studies and data from the literature showing effective antagonist activity (with receptor binding and downstream signalling pathway inhibition) (Engeroff et al., 2022; Frank et al., 2008; Gasse et al., 2007; Gu et al., 2021; Jones et al., 2014; Lindauer et al., 2009; Nadeau‐Vallée et al., 2017; Quiniou et al., 2008, 2008; Sarıoğlu et al., 2021; Terzi et al., 2022; X. Wang et al., 2021; Zhu et al., 2023). Experimental procedures and outcome measures (detailed in the following sections) were performed on D7 after the bleomycin challenge (i.e., during the acute exudative inflammatory phase) (Figure 1).

### 2.5 Physiological parameters

Mice were weighed daily to calculate body weight changes (expressed as a percentage of initial weight), in addition to the monitoring of the other potential endpoints (respiratory failure, loss of more than 30% of the initial body weight, prostration, uncontrollable pain, dehydration), according to the procedure approved by the CRCHUM Institutional Animal Care Committee. As indicated above, none of the animals reached these combined endpoints, and no mortality was observed.

### 2.6 Euthanasia

For all procedures, except those involving measurements with the epoc® Blood Analysis System (Siemens Healthineers, Oakville, Ontario, Canada), mice were euthanized on D7 after the bleomycin challenge by intraperitoneal (i.p.) injection of an overdose of pentobarbital (75 mg/kg) followed by sectioning of the inferior vena cava, as recommended by the Institutional Animal Protection Committee of the Centre de recherche du Centre hospitalier de l'Université de Montréal (CRCHUM), according to the guidelines of the Canadian Council on Animal Care (CCAC).

### 2.7 Measurements of blood pH and $P_{CO_{2}}$ with the epoc blood analysis system

To reduce the number of animals needed per procedure/outcome, in accordance with the 3Rs principles (CCAC), mice used for blood parameter measurements on D7 were also used for other analyses, in particular for lung histological assessment or inflammatory profiling after BAL sampling. On D7, mice were deeply anaesthetized with isoflurane (3% mixed with 21% O_2_ and medical air), prior to and during venous blood sampling from the vena cava using a heparinized needle and syringe for measurement of blood pH and partial pressure of carbon dioxide ($P_{CO_{2}}$) using the epoc Blood Analysis System. Deep anaesthesia was determined by the absence of hindlimb pedal withdrawal reflex and eye blink reflex. During this terminal surgery, breathing and absence of reflexes were visually monitored by the experimenter to ensure that the animal did not show signs of pain. The percentage of isoflurane was increased as necessary. Mice were then euthanized by cutting the inferior vena cava, followed by cervical dislocation, to allow for subsequent lung sampling and outcome measurements to be performed (as described below).

### 2.8 Lung water content measurement (as an index of lung oedema)

After euthanasia on D7, the lungs were removed and directly weighed (wet weight). They were then heated to 95°C for 24 h to measure the dry weight and then to calculate the lung water content (LWC) ratio ([wet weight − dry weight]/D0 body weight).

### 2.9 Bronchoalveolar lavages

In another series of experiments, BALs were performed after euthanasia on D7 post‐bleomycin challenge by three repeated intratracheal (IT) instillations of 1 mL of PBS, which were then pooled on ice. After centrifugation (700 *g*, 4°C, 8 min), the supernatant was aliquoted and stored at −80°C until further use (see below).

### 2.10 Measurements of protein concentration in BALs

Protein concentration in BAL supernatants was determined by the Bradford method (Bio‐Rad Laboratories, Mississauga, ON, Canada).

### 2.11 Total and differential immune cell counts

Cell pellets obtained after BAL centrifugation were resuspended in 400 μL of PBS, before counting the total number of cells in a haemacytometer. Cell suspensions were then diluted to an approximate density of 4 × 10^5^ cells/ml before cytocentrifugation (850 rpm (82 *g*), 6 min, Cytospin 4 Cytocentrifuge, Fisher Scientific, Runcorn, UK) onto glass slides (8 × 10^4^ cells/spot). Cells were then stained with Eosin‐Y (71225; Richard‐Allan Scientific, Kalamazoo, MI, USA) and Harris Hematoxylin (Hg free; 10143‐608; VWR, Mississauga, Ontario, CA) to allow differential cell counting (number of neutrophils reported as a percentage of a total of 400 leukocytes/slide). For a more accurate total cell count, the glass slides were scanned at ×200 (Aperio Versa 200; Leica Biosystems Inc., Concord, Canada) and then analysed using Visiopharm software (Visiopharm, Hoersholm, Denmark).

### 2.12 RNA extraction and real‐time quantitative PCR

After harvesting, the lungs were immediately snap‐frozen in 2‐methylbutane on dry ice and stored at −80°C until needed. One of the lungs was crushed in liquid nitrogen and the total RNA was extracted using the RNeasy mini kits (Qiagen, Toronto, Ontario, Canada). The RNA concentration and purity were assessed using a NanoDrop™ One Spectrophotometer (Thermo Fisher Scientific, Waltham, MA, USA). Samples without OD_260/280_ ≥ 1.8, OD_260/230_ ≥ 1.8, were disqualified. The RNA integrity was also verified by migrating the samples onto an agarose gel (1%) containing SYBR Safe DNA Gel Stain (Thermo Fisher Scientific), visualized using the Typhoon Gel Imager (Typhoon TRIO variable mode imager, GE Healthcare, Chicago, IL, USA) and the Image Lab software version 6.0.0 (Bio‐Rad Laboratories). The RNA extracts were then treated with DNase (DNA‐*free* DNA Removal Kit, Thermo Fisher Scientific) before reverse transcription of RNA (1 μg) into cDNA using the iScript Reverse Transcription SuperMix Kit (Bio‐Rad Laboratories).

For PCR amplification and quantitative analysis of IL‐6, KC (the murine equivalent of human IL‐8), TNF‐α, and MCP‐1 gene expression levels, 5 ng of cDNA was amplified using the SYBR Green Master Mix Kit (172‐5124; Bio‐Rad Laboratories) in real‐time PCR systems (Roche LightCycler (Roche Diagnostics Corp., Indianapolis, IN, USA) and Corbett Rotor‐Gene RG‐6000 Real‐Time PCR Analyzer (Qiagen Canada)) in the presence of 337.5–500 nM of forward and reverse primers designed on the National Center for Biotechnology Information Primer Blast website and synthesized by Integrated DNA Technologies (Kanata, Ontario, Canada) (see sequences in Table 1). All kits were used according to the manufacturer's instructions. The $2^{-\Delta \Delta C_{t}}$ method was used to calculate the relative expression, compared with control groups, and the expression level of the housekeeping gene 18S (AM1718; Thermo Fisher Scientific) was used for normalization.

**TABLE 1:** Primer sequences and RT‐qPCR conditions.

Gene	Primer sequences		Primer concentration	Cycling condition
IL‐6	F:	5′‐AGACAAAGCCAGAGTCCTTCAG‐3′	337.5 nM	95°C, 30 s + 40 × (95°C, 15 s + 60°C, 60 s)
	R:	5′‐TGCCGAGTAGATCTCAAAGTGA‐3′		
TNF‐α	F:	5′‐TCAGCCGATTTGCTATCTCATA‐3′		
	R:	5′‐AGTACTTGGGCAGATTGACCTC‐3′		
CCL2 (MCP‐1)	F:	5′‐AGCTGTAGTTTTTGTCACCAAGC‐3′	500 nM	95°C, 10 min + 40 × (95°C, 10 s + 60°C, 20 s + 72°C, 20 s)
	R:	5′‐GACCTTAGGGCAGATGCAGT‐3′		
CXCL1 (KC)	F:	5′‐TGAAGCTCCCTTGGTTCAG‐3′		
	R:	5′‐GGTGCCATCAGAGCAGTCT‐3′		

### 2.13 Lung collection and histological analyses

On D7 after the bleomycin challenge, the lungs were carefully inflated with 500 μL of 10% neutral formalin (ChapTec, Montréal‐Est, QC, Canada) prior to harvest, and then immersed in formalin. Fixed lung tissues were placed in 4% paraformaldehyde, dehydrated in a series of ethanol solutions of increasing concentrations (70%, 95%, 99%), cleared in xylene, embedded in paraffin, and sectioned longitudinally (4 μm) at the CRCHUM Molecular Pathology Core Facility. Lung sections were stained with Harris Hematoxylin (VWR) and Eosin‐Y (Richard‐Allan) (H&E). Because of the heterogeneity of alveolar damage between areas, even within the same animal, the entire lung section of each lung was scanned at ×200 (Aperio Versa 200) prior to blinded histological analysis by Dr Feryel Azzi (Pathologist, Molecular Pathology Platform, CRCHUM) and Dr Guillaume St‐Jean (Assistant Professor of Veterinary Anatomical Pathology, Faculty of Veterinary Medicine, Université de Montréal). Qualitative lung injury scoring was adapted from a well‐recognized scoring system used to evaluate experimental ALI in animals (Matute‐Bello et al., 2011). The severity of lung inflammation and septal wall thickening (on a scale of 1–3) and the area of the lung with evidence of inflamed/damaged foci (as a percentage of the total lung section) were assigned to each section.

### 2.14 Statistical analysis

Data are presented as means ± standard deviation (SD). Graphs and statistical analyses were performed using GraphPad Prism v. 9.3.1 software (GraphPad Software, Boston, MA, USA). Normality tests (D'Agostino–Pearson) were performed first, followed by statistical tests, adapted to each type of experiment, as indicated in each figure legend. A *P*‐value < 0.05 was considered significant. An excel file with the *P*‐value for each of the statistical test for multiple comparisons is supplied as Supporting information.

## 3 RESULTS

**FIGURE 2:**
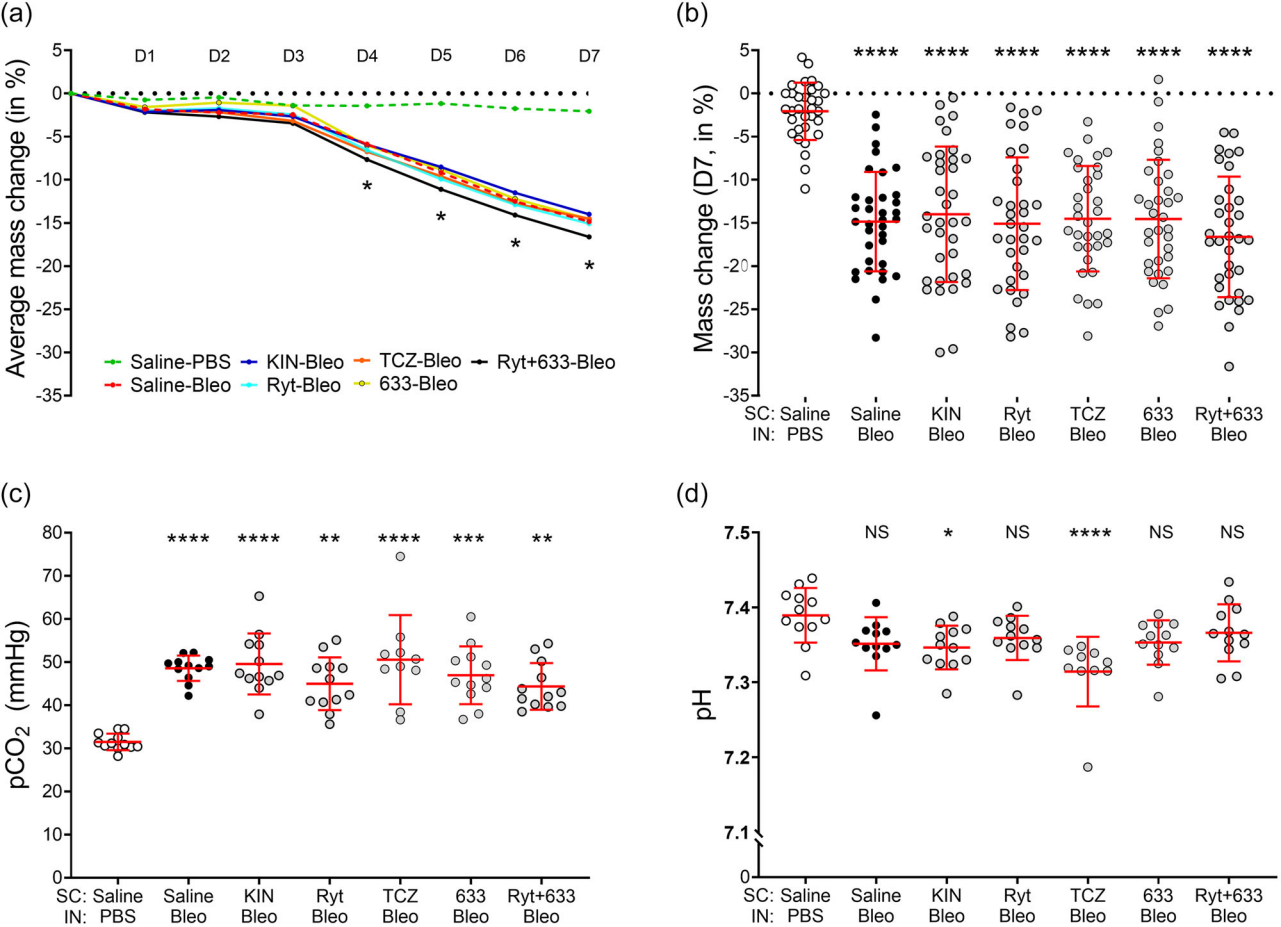
Effect of treatments with IL‐1 and IL‐6 receptor antagonists on body weight changes and pulmonary function in mice after bleomycin‐induced acute lung injury. Mice received i.n. instillation of bleomycin (Bleo, 3 U/kg) or PBS on day 0 and were injected (s.c.) twice daily with either vehicle (Saline) or IL‐1 and IL‐6 receptor antagonists (Kineret 12.5 mg/kg/dose, rytvela 1 mg/kg/dose, tocilizumab 10 mg/kg/dose, HSJ633 1 mg/kg/dose or a combination of rytvela+HSJ633) for 7 days. (a, b) Mean change in body weight over time (a) and individual mass change on day 7 (b). *n* = 32–35 per condition (each day). (c, d) Venous $P_{CO_{2}}$ (c) and pH (d) on day 7, *n* = 10–12. (a) was analysed with mixed‐effects model (restricted maximum likelihood) and Dunnett's multiple comparisons versus Saline‐PBS (*P < 0.05 for each condition) and versus Saline‐Bleo (*P* ≥ 0.05 not showed). (b) was analysed with Welch's ANOVA test and Dunnett's T3 multiple comparison test and (c, d) with Kruskal–Wallis and Dunn's multiple comparisons test. Multiple comparisons against Saline‐PBS (****P < 0.0001, ***P < 0.001, **P < 0.01, *P < 0.05, NS *P* ≥ 0.05) and against Saline‐Bleo (*P* ≥ 0.05 not shown). Circles are individual mice and red lines are means ± SD.

Before examining the effect of treatments targeting IL‐1 and IL‐6 receptors on the main features of the exudative phase of bleomycin‐induced acute lung injury (i.e., lung damage, oedema flooding and inflammatory response), we assessed the general condition of the animals in our experimental groups (*n* = 32–35 animals). While the mean body weight of the control group (Saline‐PBS) remained stable, mice instilled with bleomycin (3 U/kg; Saline‐Bleo, KIN‐Bleo, Ryt‐Bleo, TCZ‐Bleo, 633‐Bleo, and Ryt+633‐Bleo groups) gradually lost weight, resulting in a statistically significant and stable difference in mass change starting from day 4 (D4), compared with the control group (Figure 2a). On day 7 (D7), the average body mass change in the Saline‐PBS group was −2.071% (±3.316), compared to −14.850% (5.751) for Saline‐Bleo (Figure 2b). Treatment with the IL‐1Ra and IL‐6Ra in bleomicyn‐instilled mice (KIN‐Bleo, Ryt‐Bleo, TCZ‐Bleo, 633‐Bleo and Ryt+633‐Bleo) had no significant beneficial effect on mass change (Figure 2a,b). The survival rate was 100% for each group (data not shown).

Bleomycin caused a significant increase (>40%) in venous $P_{CO_{2}}$ (*n* = 10–12 per condition) in both the saline and treatment (KIN‐Bleo, Ryt‐Bleo, TCZ‐Bleo, 633‐Bleo and Ryt+633‐Bleo) groups (Figure 2c), indicating an impaired respiratory function. Mice showed signs of acidification in all bleomycin groups, although no significant difference was found between the mean of the Saline‐PBS and the Saline‐Bleo groups (Figure 2d, *n* = 10–12).

**FIGURE 3:**
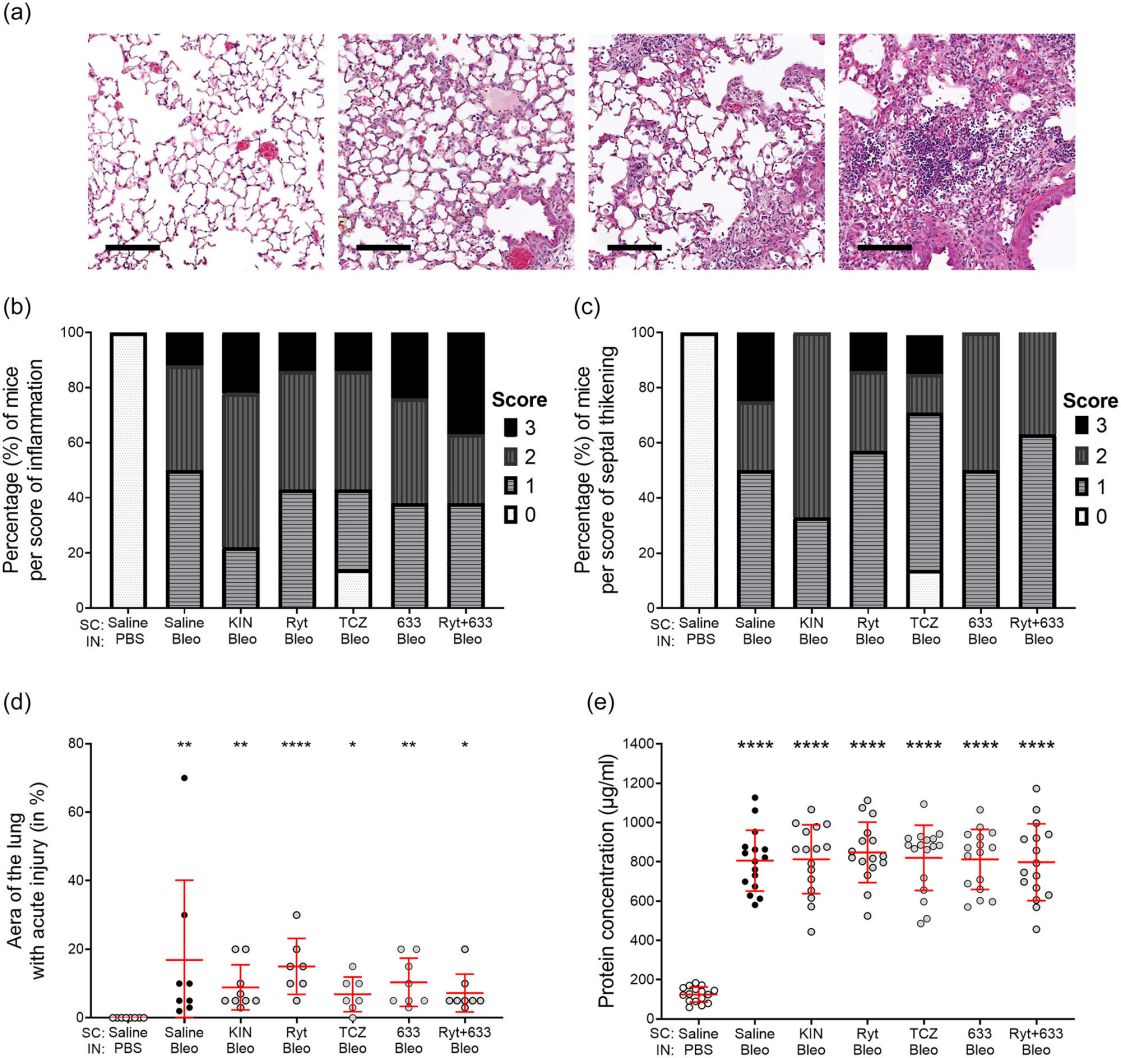
Effect of treatments with IL‐1 and IL‐6 receptor antagonists on lung inflammatory damage after bleomycin‐induced acute lung injury at D7. Mice received i.n. instillation of bleomycin 3 U/kg or PBS at D0 and were injected (s.c.) twice daily with either vehicle (Saline) or IL‐1 and IL‐6 receptor antagonists (Kineret 12.5 mg/kg/dose, rytvela 1 mg/kg/dose, tocilizumab 10 mg/kg/dose, HSJ633 1 mg/kg/dose or a combination of rytvela+HSJ633) for 7 days, *n* = 7–9. (a) Representative images of the H&E‐stained lung parenchyma for each score level (scale (black line) = 200 μm, ×200 magnification). (b) Percentage of mice per score of inflammation (×2 (18 degrees of freedom (df), *n* = 55) = 53.04, *P* < 0.0001) and (c) septal thickening (×2 (18 df, *n* = 55) = 60.14, *P* < 0.0001). (d) Percentage of zones with acute injury/inflammation per lung section, Kruskal–Wallis and Dunn's multiple comparison test. (e) Protein concentration in BALs, indicator of alveolar injury (Welch's ANOVA, Dunn's T3 multiple comparisons test, *n* = 16–17). Multiple comparisons versus Saline‐PBS (****P < 0.0001, ***P < 0.001, **P < 0.01, *P < 0.05) and versus Saline‐Bleo (*P* ≥ 0.05 not shown). Circles are individual mice and red lines are means ± SD.

Blinded, qualitative histological analyses of lung tissues collected at D7 (*n* = 7–9), based on a pathologist‐defined scoring system (Figure 3a), revealed the presence of diffuse pulmonary injury in the vast majority of the bleomycin‐challenged mice, with an inflammatory infiltrate (predominantly neutrophils and some lymphocytes, Figure 3b) and septal thickening (Figure 3c), whereas control (Saline‐PBS) mice showed no evidence of injury. Approximately 5%–20% of the total lung area of bleomycin‐challenged mice showed foci of inflammatory damage (Figure 3d). No apparent beneficial effect of IL‐1Ra and IL‐6Ra treatments on the injury scores was observed (Figure 3b,d).

While protein concentration in BALs (Figure 3e, *n* = 16–17), an indicator of lung injury, was ∼6.5‐fold higher after the bleomycin challenge, none of the treatments with IL‐1β and IL‐6 receptor antagonists attenuated this increase.

**FIGURE 4:**
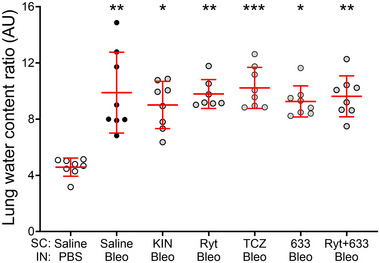
Effect of treatments with IL‐1 and IL‐6 receptor antagonists on lung oedema after bleomycin‐induced acute lung injury at D7. Mice received an i.n. instillation of bleomycin 3 U/kg or PBS at D0 and were injected (s.c.) twice daily with either vehicle (Saline) or IL‐1 and IL‐6 receptor antagonists (Kineret 12.5 mg/kg/dose, rytvela 1 mg/kg/dose, tocilizumab 10 mg/kg/dose, HSJ633 1 mg/kg/dose or a combination of rytvela+HSJ633) for 7 days. Kruskal–Wallis test, Dunn's multiple comparison test, *n* = 7‐8. Multiple comparisons versus Saline‐PBS (***P < 0.001, **P < 0.01, *P < 0.05) and versus Saline‐Bleo (*P* ≥ 0.05 not showed). Circles are individual mice and red lines are means ± SD.

Similarly, the different treatments did not prevent the development of pulmonary oedema, induced by bleomycin, as shown by the measurements of LWC ratio in all groups tested (Figure 4, *n* = 7–8).

**FIGURE 5:**
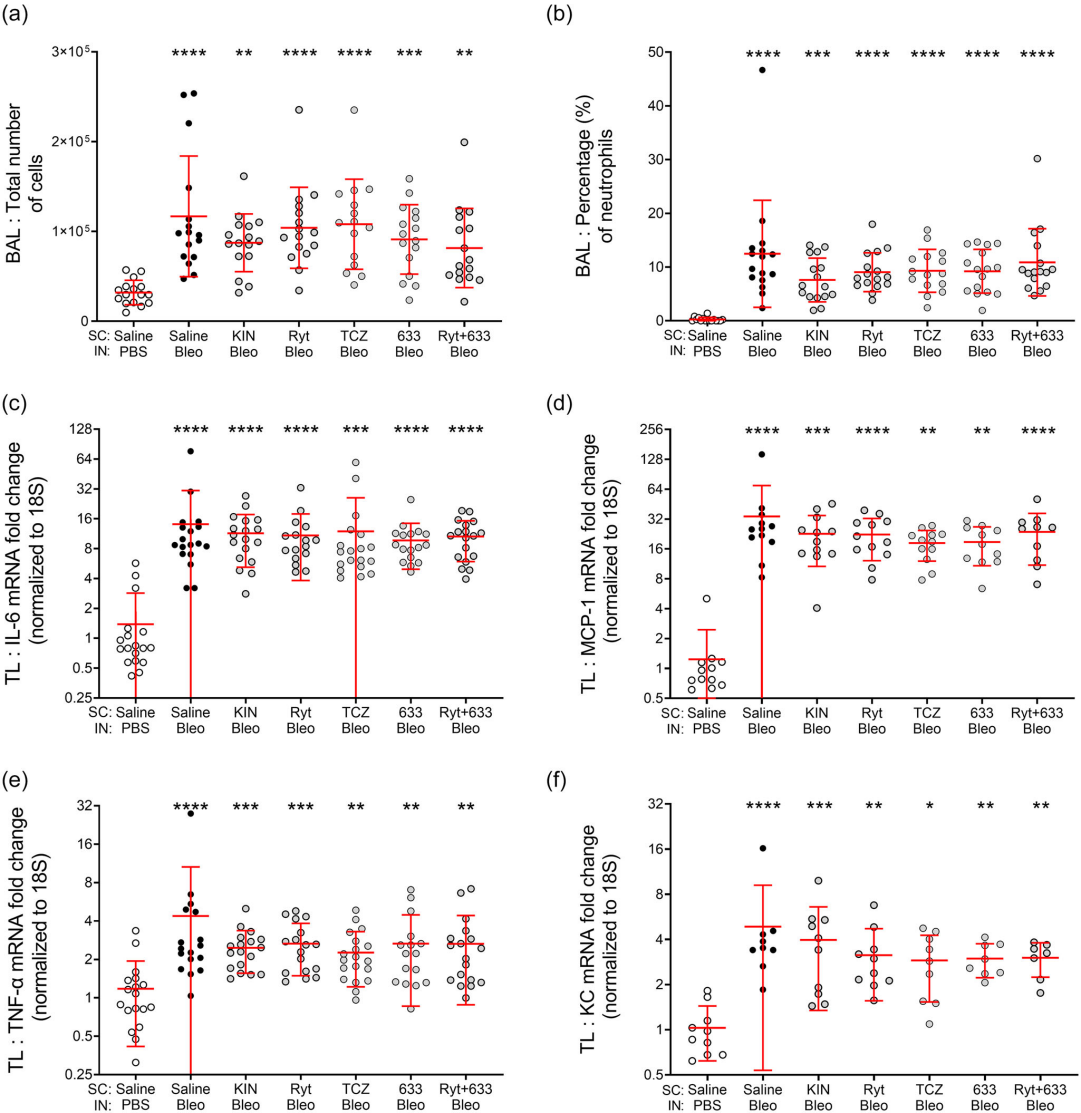
Effect of treatments with IL‐1 and IL‐6 receptor antagonists on the inflammatory response after bleomycin‐induced acute lung injury in mice at D7. Mice received an i.n. instillation of bleomycin 3 U/kg or PBS at D0 and were injected (s.c.) twice daily with either vehicle (Saline) or IL‐1 and IL‐6 receptor antagonists (Kineret 12.5 mg/kg/dose, rytvela 1 mg/kg/dose, tocilizumab 10 mg/kg/dose, HSJ633 1 mg/kg/dose or a combination of rytvela+HSJ633) for 7 days. (a, b) Total number of cells (a) and percentage of neutrophils (b) in BALs, *n* = 15–16. (c–f) mRNA (from total lung (TL)) fold changes normalized to 18S for IL‐6 (c) (*n* = 16‐19), MCP‐1 (d) (*n* = 10–12), TNF‐α (e) (*n* = 16–19), KC (f) (*n* = 7–10). Data set were analysed using Kruskal–Wallis and Dunn's multiple comparison test. Multiple comparisons versus Saline‐PBS (***P < 0.001, **P < 0.01, *P < 0.05, NS *P* ≥ 0.05) and versus Saline‐Bleo (*P* ≥ 0.05 not shown). Circles are individual mice and red lines are means ± SD.

Finally, the effect of the IL‐1β and IL‐6 receptor antagonists on the bleomycin‐induced inflammatory response was evaluated. We observed that the total number of infiltrating cells was increased after the bleomycin challenge (by 3.7‐fold), as well as the proportion of neutrophils in BALs (Figure 5a,b, *n* = 15‐16). Treatments targeting IL‐1β and IL‐6 receptors did not show a significant improvement in these parameters.

Bleomycin instillation also induced an increase in pro‐inflammatory cytokine/chemokine mRNA levels in lung tissue extracts (Figure 5c–f), as indicated by quantitative real‐time RT‐qPCR measurements of MCP‐1 (a macrophage chemoattractant; *n* = 10–12), KC (the murine equivalent of IL‐8; a neutrophil chemoattractant; *n* = 7–10), TNF‐α (*n* = 16–19) and IL‐6 (*n* = 16–19). Treatment with the receptor antagonists did not significantly reduce these pro‐inflammatory molecules. Similarly, ELISAs on BAL proteins indicated that the increase in IL‐6 and MCP‐1 levels was not prevented by IL‐1β and IL‐6 receptor antagonists (Supplementary Figure 1).

## 4 DISCUSSION

Our results showed that the administration of IL‐1R and IL‐6R antagonists failed to prevent body weight loss, improve lung integrity and oedema resolution, and reduce the inflammatory response in bleomycin‐challenged mice at day 7.

As expected, bleomycin instillation resulted in a significant loss of body weight, when compared to the control group. Data from the literature indicate a relationship between bleomycin dose and the severity of acute lung injury (e.g., estimated by the degree of pulmonary oedema) and between bleomycin dose and body weight loss (Gilhodes et al., 2017), suggesting a link between weight loss and the magnitude of lung injury. Actually, bleomycin debilitation is associated with a general deterioration in body condition scoring, with systemic loss of muscle and fat, reduced food intake and physical activity. Our data also showed that treatment with IL‐6Ra and/or IL‐1Ra did not alleviate the observed body weight loss (Figure 2a,b). In a murine model of LPS‐induced ALI, Kineret was previously shown, at an early time point (day 2), to reduce weight loss, which was, however, no longer different from untreated LPS mice by day 7 (Engeroff et al., 2022). Similarly, we previously reported that the glucocorticoid dexamethasone failed to prevent the bleomycin‐induced weight loss over a 7‐day period (Aubin Vega et al., 2019).

A large proportion of bleomycin‐challenged mice (but not control mice) exhibited an increase in $P_{CO_{2}}$ and an acidification of pH, indicating a state of respiratory acidosis (Figure 2c,d). Likewise, a recent paper reported blood gas analyses indicative of hypercapnia, hypoxemia and acidosis, occurring on day 7 after bleomycin (Kitzerow et al., 2022). Our measurements also showed that treatment with the different compounds did not ameliorate the observed respiratory acidosis observed in bleomycin‐challenged mice. In experimental studies of ALI induced by LPS and/or mechanical ventilation (ventilator‐induced lung injury), blood gas analysis has shown conflicting results, with either an improvement in hypoxaemia (Frank et al., 2008; Jones et al., 2014) or a lack of benefit on O_2_ saturation in the study by X. Wang et al. (2021).

Our results demonstrated that the IL‐1Ra and IL‐6Ra were ineffective in preventing lung injury, as evidenced by histopathological scoring of inflammatory damage and BAL protein concentration (Figure 3). In some other models of ALI (induced by OA, LPS or sepsis), beneficial effects of tocilizumab on lung injury scores have been observed (Sarıoğlu et al., 2021; Terzi et al., 2022; Zhu et al., 2023). While the literature reports a lack of effect of Kineret on histopathological scores and lavage protein levels in an animal model of mechanical ventilation combined with LPS (Jones et al., 2014; X. Wang et al., 2021), a significant decrease in BAL protein concentration was observed in an inflammatory model induced by LPS alone (Engeroff et al., 2022). We have also previously shown that rytvela is more effective than Kineret in preventing the lung injury observed in adolescent mice following antenatal IL‐1β injection and that it is beneficial in promoting lung tissue integrity in both IL‐1β‐ and LPS‐induced preterm birth models (Habelrih et al., 2023; Nadeau‐Vallée et al., 2017).

LWC ratios show no improvement in the resolution of oedema in groups of bleomycin mice treated with IL‐1Ra and IL‐6Ra (Figure 4). These data are inconsistent with what Zhu et al. (2023) obtained with a single i.p. injection of tocilizumab (4 mg/kg) in a rat caecal ligation and puncture (CLP) model or to what Frank et al. (2008) observed in a rat model of ventilator‐induced lung injury with a loading dose of intra‐jugular IL‐1Ra (10 mg/kg) prior to mechanical ventilation, followed by a continuous infusion (10 mg/kg/h) throughout the protocol. Therefore, differences in protocols, doses and models may explain these conflicting results.

Analyses of the bleomycin‐induced inflammatory response, by measuring immune cell counts in BALs and cytokine levels in lung tissue showed, as expected, a rise in immune cell counts and neutrophil infiltration as well as an increase in MCP‐1, KC, TNF‐α and IL‐6 mRNA at D7 in all groups of bleomycin‐challenged mice (Figure 5). Our protein analysis in BALs also confirmed an increase in IL‐6 and MCP‐1 levels (Supplementary Figure 1). Although a downward trend was observed for some parameters, none of the IL‐6Ra and IL‐1Ra compounds produced a statistically significant effect on this inflammatory response. Evidence from the literature regarding the potential anti‐inflammatory effects of tocilizumab (Gu et al., 2021; Sarıoğlu et al., 2021; Terzi et al., 2022; Zhu et al., 2023) and Kineret (Frank et al., 2008; Gu et al., 2021; Jones et al., 2014; Lindauer et al., 2009; X. Wang et al., 2021) in various animal models of ALI is conflicting and varies depending on the experimental conditions. Analysis of short and long time points (day 1 and 14) after bleomycin (15 mg/kg at D0) in mice treated with Kineret (1 mg/kg) previously showed a reduction in neutrophil infiltration (at D1 and at D14) and pro‐inflammatory cytokines (at D1, KC, IL‐6, IL‐1β) (Gasse et al., 2007).

In summary, our study did not show a beneficial effect of the IL‐1Ra and IL‐6Ra we tested, and analysis of the literature reflects a lack of consensus regarding the ability of tocilizumab and Kineret to improve key parameters of ALI. Nevertheless, the overall evidence from the literature suggests that in animal models of ALI where inflammation is the primary source of injury (LPS, Poly I:C + spike), beneficial effects of tocilizumab and/or Kineret on at least some of the parameters of ALI have been reported (Engeroff et al., 2022; Gu et al., 2021; Sarıoğlu et al., 2021). Similarly, in a recent clinical trial, ferritin, an inflammatory biomarker, was positively associated with better clinical outcomes with tocilizumab treatment, compared to placebo (Tom et al., 2022). As reviewed in Fadanni & Calixto (2023), clinical trials and observational studies (particularly on COVID‐19 ARDS) have shown that treatment with IL‐1Ra and IL‐6Ra is more beneficial in the severe form of the disease, in patients with hyper‐inflammatory phenotypes. Indeed, analysis of clinical and biological heterogeneity has revealed distinct phenotypes defined as either hypo‐ or hyper‐inflammatory in ARDS patients (Bos & Ware, 2022; Calfee et al., 2015; Reddy et al., 2020; H. Xu et al., 2023). It is becoming increasingly clear that the heterogeneity of ARDS is largely responsible for the lack of efficacy of most treatments in clinical trials. Therefore, patient phenotypes may be associated not only with variability in outcome severity, but also with inconsistent responses to ARDS management and treatments (Battaglini et al., 2023; Beitler et al., 2022; Reddy et al., 2020; Rizzo et al., 2023; H. Xu et al., 2023).

When the primary cause of the observed lung structural damage is not directly due to the deleterious effects of inflammatory cytokines such as IL‐6 and IL‐1β, drugs that act on IL‐6 or IL‐1 receptors may be ineffective. Therefore, in animal models that exhibit extensive lung damage and pulmonary oedema in addition to inflammation, thus mimicking the key features of ARDS (Matute‐Bello et al., 2008; Monahan, 2013), there is, to the best of our knowledge, no clear evidence of beneficial effects of IL‐6R or IL‐1R antagonists at the peak of the acute exudative phase of ALI (day 7), which is a critical time point for ARDS resolution (Matthay et al., 2019; Ware & Matthay, 2000). The bleomycin‐induced ALI model is one such example, as bleomycin causes direct alveolar tissue damage by inducing DNA breakage, free radical production and ultimately cell death (at least in part via ferroptosis) (Burger et al., 1981; Matute‐Bello et al., 2008; Reinert et al., 2013; Zhan et al., 2022), prior to immune cell infiltration/activation. Thus, the bleomycin model can mimic ALI/ARDS from a direct intrapulmonary insult, but not from an infectious origin, which is associated with an early inflammatory response. The lack of beneficial effects of IL‐1 and IL‐6 receptor antagonists observed in our study is consistent with the fact that although IL‐1β and IL‐6 are produced, these cytokines may not be the primary contributors to the observed damage at D7. Furthermore, in a previous study, we showed that targeting inflammation, with a broad‐spectrum anti‐inflammatory drug (dexamethasone), failed to improve bleomycin outcomes, particularly mortality, immune cell infiltration and pulmonary oedema, likely due to residual alveolar damage and impairment of repair processes (Aubin Vega et al., 2019). Due to the importance of the lesional component of ARDS (Butt et al., 2016; Matthay et al., 2019; Monahan, 2013), the development of novel therapeutic approaches that promote the restoration of alveolar integrity and function, for example, with mesenchymal stromal cells (Wick et al., 2021) or by targeting key proteins involved in epithelial repair processes, is critical for ARDS recovery, before the establishment of irreversible fibrosis. Compelling evidence suggests that a class of membrane proteins, that is, K^+^ channels, play an important role in epithelial repair and play a protective role in animal models of epithelial injury (Abe et al., 2020; Arni et al., 2021; Girault & Brochiero, 2014; He et al., 2019; Jia et al., 2022; Schwingshackl et al., 2017; Trinh et al., 2007, 2008; Zhou et al., 2019; Zyrianova et al., 2020). KvLQT1 and K_ATP_ K^+^ channels also regulate alveolar fluid clearance in vitro and in vivo (Abe et al., 2020; Aubin Vega et al., 2023; Bardou et al., 2012; Leroy et al., 2006; Sakuma et al., 1998; Zhou et al., 2019). Thus, such strategies that favour the resorption of pulmonary oedema and the restoration of epithelial integrity, by targeting ion channels, combined with anti‐inflammatory treatments, may be beneficial for the resolution of ARDS. Although negative results were obtained with IL‐1R and IL‐6R modulators in our model of ALI induced by bleomycin, further studies, in complementary models of ALI, from direct and indirect causes, with either hypo‐ or hyper‐inflammatory phenotypes, may be useful to evaluate the efficacy of anti‐cytokine drugs in combination with pro‐regenerative strategies. The identification of novel treatable traits and therapeutic targets, as well as innovative human models, to predict the response to therapies, adapted to patient subphenotypes, is critical to pave the way for effective personalized medicine in ARDS.

## AUTHOR CONTRIBUTIONS

Emmanuelle Brochiero, Sylvain Chemtob, Émilie Meunier, Mélissa Aubin Vega, Damien Adam and Christiane Quiniou were involved in the conception and design of the study. Émilie Meunier, Mélissa Aubin Vega, Anik Privé, and Mohammad Ali Mohammad Nezhady participated in data acquisition (at the Centre de Recherche du Centre Hospitalier de l'Université de Montréal (CRCHUM) and at the Centre de recherche du Centre hospitalier Universitaire Sainte‐Justine). Data analyses and interpretation was performed by Émilie Meunier, Mélissa Aubin Vega, Damien Adam, Mohammad Ali Mohammad Nezhady, Isabelle Lahaie, Christiane Quiniou, Sylvain Chemtob and Emmanuelle Brochiero. Émilie Meunier and Emmanuelle Brochiero drafted the manuscript, all other authors participated in the critical revision of the article. All authors have read and approved the final version of this manuscript and agree to be accountable for all aspects of the work in ensuring that questions related to the accuracy or integrity of any part of the work are appropriately investigated and resolved. All persons designated as authors qualify for authorship, and all those who qualify for authorship are listed. The authors declare that this manuscript does not contain artificial intelligence generated content.

## CONFLICT OF INTEREST

S.C. and C.Q. hold a patent on a composition of matter for the use of 101.10 (rytvela; interleukin 1 receptor antagonist, compositions, and methods of treatment; US Patent number USPTO8618054; May 5, 2005). The remaining authors declare that the research was conducted in the absence of any commercial or financial relationship that could be construed as a potential conflict of interest.

## Supporting information

Supplementary Figure.

Appendix *P* values.

## Data Availability

The data that support the findings of this study are available from the corresponding author upon reasonable request.
