# Supplementary material for: Evaluation of interleukin‐1 and interleukin‐6 receptor antagonists in a murine model of acute lung injury
Source: Exp Physiol. 2024 Apr 9;109(6):966–79. doi: 10.1113/EP091682 (PMC11140168; doi:10.1113/EP091682)
Supplement: Supplementary file 1 — Supplementary Figure. [file EPH-109-966-s002.pdf]

Fig 1

	Mass change for each day (fig. 1A)						
	Day 1	Day 2	Day 3	Day 4	Day 5	Day 6	Day 7
Saline-PBS vs. Saline-Bleo	0,187	0,046	0,507	<0,0001	<0,0001	<0,0001	<0,0001
Saline-PBS vs. KIN-Bleo	0,019	0,047	0,314	0,001	<0,0001	<0,0001	<0,0001
Saline-PBS vs. Ryt-Bleo	0,211	0,277	0,723	0,002	<0,0001	<0,0001	<0,0001
Saline-PBS vs. TCZ-Bleo	0,086	0,022	0,084	<0,0001	<0,0001	<0,0001	<0,0001
Saline-PBS vs. 633-Bleo	0,275	0,804	>0,9999	<0,0001	<0,0001	<0,0001	<0,0001
Saline-PBS vs. Ryt+633-Bleo	0,041	0,003	0,048	<0,0001	<0,0001	<0,0001	<0,0001
Saline-Bleo vs. KIN-Bleo	0,968	0,991	0,9996	>0,9999	0,992	0,963	0,991
Saline-Bleo vs. Ryt-Bleo	0,9996	0,962	>0,9999	0,994	0,988	0,9996	0,9998
Saline-Bleo vs. TCZ-Bleo	0,9996	0,9999	0,902	0,942	0,998	0,9997	0,9997
Saline-Bleo vs. 633-Bleo	0,993	0,297	0,627	0,9998	0,9996	0,9997	0,9997
Saline-Bleo vs. Ryt+633-Bleo	0,966	0,961	0,736	0,496	0,466	0,716	0,728

	Mass change D7 (fig. 1B)		pCO_2 (fig. 1C)		pH (fig. 1D)	
	Saline PBS	Saline Bleo	Saline PBS	Saline Bleo	Saline PBS	Saline Bleo
Saline Bleo	<0,0001		<0,0001		0,0971	
KIN Bleo	<0,0001	0,996	<0,0001	>0,9999	0,0211	>0,9999
Ryt Bleo	<0,0001	>0,9999	0,0033	0,8313	0,4349	>0,9999
TCZ Bleo	<0,0001	>0,9999	<0,0001	>0,9999	<0,0001	0,1116
633 Bleo	<0,0001	>0,9999	0,0002	>0,9999	0,1355	>0,9999
Ryt+633 Bleo	<0,0001	0,8219	0,007	0,5543	0,6648	>0,9999

Fig 2

	Aera of the lung with acute injury (fig. 2D)		Protein concentration (fig. 2E)	
	Saline PBS	Saline Bleo	Saline PBS	Saline Bleo
Saline Bleo	0,0036		<0,0001	
KIN Bleo	0,0039	>0,9999	<0,0001	>0,9999
Ryt Bleo	<0,0001	>0,9999	<0,0001	0,9663
TCZ Bleo	0,0462	>0,9999	<0,0001	>0,9999
633 Bleo	0,0018	>0,9999	<0,0001	>0,9999
Ryt+633 Bleo	0,0242	>0,9999	<0,0001	>0,9999

Fig 3

	Water-lung-content ratio (fig. 3)	
	Saline PBS	Saline Bleo
Saline Bleo	0,0087	
KIN Bleo	0,0182	>0,9999
Ryt Bleo	0,001	>0,9999
TCZ Bleo	0,0003	>0,9999
633 Bleo	0,0173	>0,9999
Ryt+633 Bleo	0,0024	>0,9999

Fig 4

	Total number of cells (fig. 4A)		Percentage of neutrophils (fig. 4B)		MCP-1 mRNA fold change (fig. 4C)		KC mRNA fold change (fig. 4D)		TNF-α mRNA fold change (fig. 4E)		IL-6 mRNA fold change (fig. 4F)	
	Saline PBS	Saline Bleo	Saline PBS	Saline Bleo	Saline PBS	Saline Bleo	Saline PBS	Saline Bleo	Saline PBS	Saline Bleo	Saline PBS	Saline Bleo
Saline Bleo	<0,0001		<0,0001		<0,0001		<0,0001		<0,0001		<0,0001	
KIN Bleo	0,0012	0,2272	0,0007	0,4725	0,0001	>0,9999	0,0007	>0,9999	0,0002	>0,9999	<0,0001	>0,9999
Ryt Bleo	<0,0001	0,658	<0,0001	>0,9999	<0,0001	>0,9999	0,0059	>0,9999	0,0002	>0,9999	<0,0001	>0,9999
TCZ Bleo	<0,0001	0,658	<0,0001	>0,9999	0,0023	0,9437	0,0148	>0,9999	0,0062	>0,9999	0,0002	>0,9999
633 Bleo	0,0008	0,2784	<0,0001	>0,9999	0,0022	>0,9999	0,006	>0,9999	0,0051	>0,9999	<0,0001	>0,9999
Ryt+633 Bleo	0,002	0,124	<0,0001	>0,9999	<0,0001	>0,9999	0,0086	>0,9999	0,0025	>0,9999	<0,0001	>0,9999
